# CDK5 Is Essential for Soluble Amyloid β-Induced Degradation of GKAP and Remodeling of the Synaptic Actin Cytoskeleton

**DOI:** 10.1371/journal.pone.0023097

**Published:** 2011-07-29

**Authors:** Francesco Roselli, Paolo Livrea, Osborne F. X. Almeida

**Affiliations:** 1 Neuroadaptation Group, Max Planck Institute of Psychiatry, Munich, Germany; 2 Department of Neurological and Psychiatric Sciences, University of Bari, Bari, Italy; Federal University of Rio de Janeiro, Brazil

## Abstract

The early stages of Alzheimer's disease are marked by synaptic dysfunction and loss. This process results from the disassembly and degradation of synaptic components, in particular of scaffolding proteins that compose the post-synaptic density (PSD), namely PSD95, Homer and Shank. Here we investigated in rat frontal cortex dissociated culture the mechanisms involved in the downregulation of GKAP (SAPAP1), which links the PSD95 complex to the Shank complex and cytoskeletal structures within the PSD. We show that Aβ causes the rapid loss of GKAP from synapses through a pathway that critically requires cdk5 activity, and is set in motion by NMDAR activity and Ca^2+^ influx. We show that GKAP is a direct substrate of cdk5 and that its phosphorylation results in polyubiquitination and proteasomal degradation of GKAP and remodeling (collapse) of the synaptic actin cytoskeleton; the latter effect is abolished in neurons expressing GKAP mutants that are resistant to phosphorylation by cdk5. Given that cdk5 also regulates degradation of PSD95, these results underscore the central position of cdk5 in mediating Aβ-induced PSD disassembly and synapse loss.

## Introduction

In the early stages of Alzheimer's disease (AD), soluble oligomers of amyloid-β (Aβ) bind to synaptic sites, derange synaptic plasticity and ultimately cause the loss of synapses [1]–[4]. Decrease in synapse number and the ensuing derangement in brain connectivity are the best predictors of the onset and the progression of cognitive impairment in AD [5], [6]. While both the pre- and post-synaptic compartments are affected by Aβ peptides [7], loss of dendritic spines is the most prominent effect of Aβ observed in transgenic animals [8] and in neurons in culture [9]–[11]. Sequentially interconnected molecular events underlie the process leading to spine demise upon exposure to Aβ: endocytosis of synaptic AMPA and NMDA glutamate receptors [9], [10], [12], [13], retro-translocation of adhesion molecules and kinases [10], [14], and depolymerization of actin structures [10], [14], [15].

Major reorganization of the scaffold proteins that constitute the post-synaptic density (PSD) [16] is observed: PSD-95 undergoes degradation after phosphorylation by cdk5, and activation of multiple signaling pathways lead to the dispersal of Homer1 and Shank1 clusters [13], [17]. Since the PSD plays a pivotal role in the formation and maintenance of spines [16], Aβ-induced PSD disassembly is likely to represent a point beyond which synaptic loss becomes inevitable.

GKAP/SAPAP family proteins serve a crucial function in the organization of the PSD by effectively bridging PSD-95 and Shank complexes and thus, bringing glutamate receptors (part of the PSD-95 complex), cytoskeletal and signaling (partners of Shank proteins) proteins in close proximity of each other [18]–[21]. The loss of SAPAP interferes with the ultrastructural organization of the PSD and maturation and plasticity of synapses [22]. GKAP, the shortest and most abundant SAPAP1 isoform [18], [23], is located in the deepest layer of the PSD [24] where, in addition to PSD-95 and Shank, it directly interacts with cytoskeletal structures [25]–[27]. Given that GKAP resides at an interface where different scaffold modules (the PSD and the synaptic cytoskeleton) interact, understanding how GKAP is regulated by Aβ can be expected to provide critical insights into how amyloid peptides lead to PSD disassembly and cytoskeletal derangement. In this investigation of the fate and the regulation of GKAP by soluble Aβ peptide, we identify a crucial role of cdk-5 in triggering GKAP ubiquitination and degradation and, in turn, a major role of cdk5-GKAP signaling in the disassembly of synaptic actin structures.

## Results

### Aβ peptides disassembles synaptic GKAP clusters

**Figure 1:**
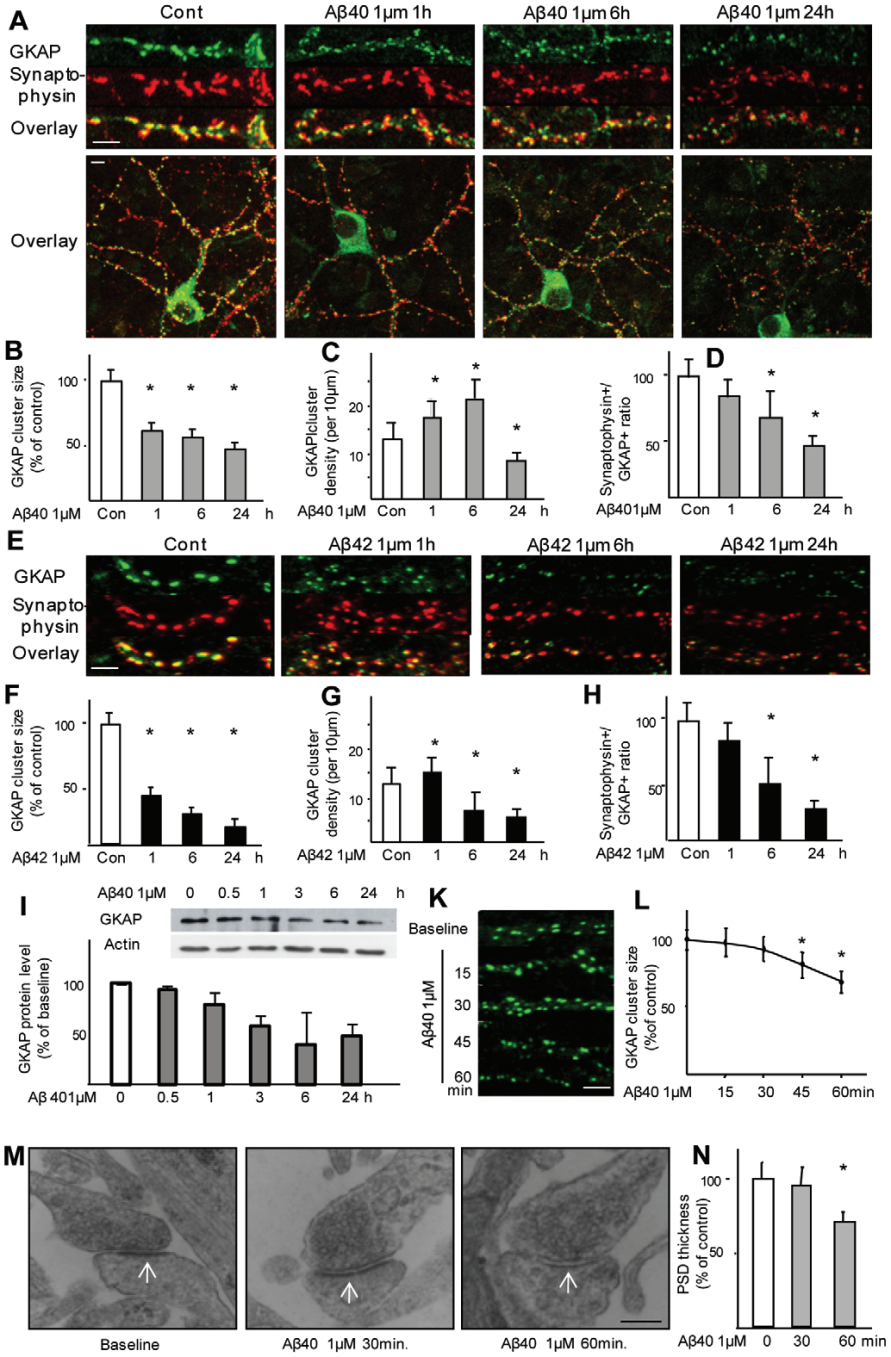
Aβ causes the downregulation of GKAP. (*A,B*) Rat frontal cortical neurons were treated with soluble Aβ40 (1 µM) for 1, 6, or 24 h and immunostained for GKAP and the synaptic marker synaptophysin; a minimum of 600 synapses per timepoint were evaluated. GKAP cluster size was decreased to 59±9.3% (p<0.01) of baseline after 1 h, and to 54.4±1.9 and 43.±5.4% after 6 and 24 h, respectively. Scale bars represent 5 µM for the high magnification images and 20 µM for the low-magnification overview. (C,D) The density of GKAP clusters, albeit smaller in size, increased from 12.4±4 clusters/10 µm to 18.5±3.5 and 21.1±5.3 after 1 and 6 h, but significantly decreased to 7.3±2.1 after 24 h; likewise, the number of synaptophysin puncta not matched with a GKAP puncta (an estimate of the number of synapses in which GKAP levels were undetectable) decreased to 82.5±7.7% of the baseline after 1 h and to 67.3±12.1% and 47.2±4.4% after 6 and 24 h, respectively; p<0.001. (E,F) Rat fronto-cortical neurons were treated with soluble Aβ42 (1 µM) oligomers for 1, 6, or 24 h and immunostained for GKAP and the synaptic marker synaptophysin; a minimum of 600 synapses per timepoint were evaluated. Aβ42 oligomers decreased the size of GKAP clusters to 47.4%±4.4, 34.9±1.9% and 23.5±2.4% of baseline after 1, 6 and 24 h, respectively; scalebar 5 µm. (G,H) Aβ42 oligomers caused an apparent increase in GKAP cluster density (due to fragmentation of large clusters) after 1 h of treatment (12.4±4 clusters per 10 µm of dendrite length at baseline vs 15.3±3.7 at 1 h), followed by a sharp decrease in GKAP cluster density (6.4±4.3 and 5.2±1.1 at 6 and 24 h, respectively; the percentage of synaptophysin puncta not matched by GKAP-positive puncta was also reduced (to 80.5±5.7%, 57.3±10.1% and 36.2±5.4%) by treatment with Aβ42 after 1, 6 and 24 h, respectively. (I) Aβ induces the down-regulation of GKAP protein levels. Rat frontal cortical neurons were treated with Aβ 1 µM for 0.5, 1, 3, 6 or 24 h and GKAP levels were measured by immunoblot of whole cell lysates. Significant decrease was observed at 1 h (82.3±14.3%, p<0.05) and at 3, 6, and 24 h timepoints (69.3±14.2%; and 48.4±21.3% 50.3±14.3% respectively, p<0.05). (K,L) Rat frontal neurons were treated with Aβ (1 µM) for 15, 30, 45 or 60 min before fixation and immunostaining for GKAP and synaptophysin. Aβ did not affect GKAP cluster size after 15 and 30 min of treatment (97.5±10.3% and 93.3±10.4%, both p>0.05), but significantly decreased GKAP cluster size at 45 and 60 min timepoints (81.4±9.5% and 68.6±8.5%, p<0.05). (*M,N*) Changes in PSD ultrastructure are detectable between 30 and 60 minutes after Aβ exposure. PSD was identified in EM images as an electron-dense band in the post-synaptic terminal (arrows). A minimum number of 50 synapses out of three replicates per condition was assessed. PSD thickness was comparable at baseline and after 30 min of treatment (37±4 nm and 35±4 nm), but was significantly reduced at 60 min (27±3 nm, p<0.05). Scale bar 200 nm.

To investigate the effect Aβ1–40 peptide on the scaffold protein GKAP, dissociated cultures of frontal cortical neurons were treated with soluble Aβ40 peptide; under the conditions used, Aβ40 was detectable in the culture medium largely as low-n oligomers with MW ranging from 4 to 16 KDa (monomer to tetramers: Figure S1). Synapses were identified by the presynatic marker synaptophysin, and levels of GKAP were evaluated by immunostaining. At baseline, GKAP displayed an intense, punctate staining along dendrites with 87.4% (±8.3) of GKAP clusters juxtaposed to synaptophysin-positive puncta. The size of GKAP synaptic clusters progressively decreased to 59.4%, 54.4±1.9% and 43.5±5.4% of baseline following exposure to Aβ40 for 1, 6 and 24 h, respectively (Fig. 1 A, B), in a dose-dependent manner (Figure S2). Although smaller in size, GKAP cluster density was apparently increased after 1 and 6 h of treatment (from 12.4±4 clusters per 10 µm of dendrite length to 18.5±3.5 and 21.1±5.3 after 1 and 6 h, respectively; Fig. 1C) but was markedly decreased after 24 h (7.3±2.1 clusters/10 µm). At closer inspection, whereas single GKAP clusters were juxtaposed to synaptophysin puncta at baseline, multiple small GKAP clusters were found to face single synaptophysin cluster at the 1 and 6 h timepoints, suggesting that the small clusters were remnants of larger PSD. Confirming the overall decrease in GKAP caused by Aβ, the percentage of synapses (identified by synaptophysin immunostaining) containing detectable levels of GKAP fell progressively (82.5±7.7%, 67.3±12.1% and 47.2±4.4%, respectively; p<0.001) (Fig. 1A, D). Of note, 10–15% of neurons in our cultures were not affected by Aβ (in line with what previously reported, see [10]). Hippocampal neurons treated with oligomers of Aβ1–42 for 1,6 and 24 h displayed larger decreases in GKAP cluster size (respectively, 47.4%±4.4, 34.9±1.9% and 23.5±2.4% of baseline; see Fig. 1 E, F). Notably, exposure to Aβ42 produced a small increase in GKAP cluster density (from 12.4±4 to 15.3±3.7 clusters per 10 µm of dendrite length) after 1 h; thereafter, GKAP size density was markedly reduced (6.4±4.3 and 5.2±1.1 at 6 and 24 h, respectively; Fig. 1G). Likewise, the relative number of GKAP-positive synapses were reduced to 80.5±5.7%, 57.3±10.1% and 36.2±5.4% after 1, 6 and 24 h of treatment with Aβ42 (Fig. 1H). The downregulation of GKAP by Aβ was observed in whole-cell protein extracts by immunoblotting. GKAP levels were not decreased (94.4±4.0% of baseline) after 30 min of treatment, but a small decrease was observed at 1 h (82.3±14.3%, p<0.05) and significant downregulation was seen after 3, 6, and 24 h (69.3±14.2%; and 48.4±21.3% and 50.3±14.3% respectively, p<0.05; Fig. 1E).

Inspection of the temporal pattern of synaptic GKAP loss within the first hour of exposure to Aβ revealed that, while GKAP cluster size was unchanged at 15 and 30 min, GKAP cluster sizes were significantly reduced from baseline size after 45 (81.4±9.5%) and 60 min (68.6±8.5%) (p≤0.05; Fig. 1F,G). Similar temporal dynamics of Aβ-induced disassembly of the PSD was observed by ultrastructural analyses (Fig. 1H): as compared to baseline measures (37±4 nm), PSD thickness was unaltered at 30 min (35±4 nm) but significantly reduced at 60 min (27±3 nm; p<0.05) after Aβ application. Thus, both downregulation of synaptic GKAP and the collapse of the PSD occur within 30–60 min of exposure to Aβ.

### NMDAR activity is required for GKAP downregulation

**Figure 2:**
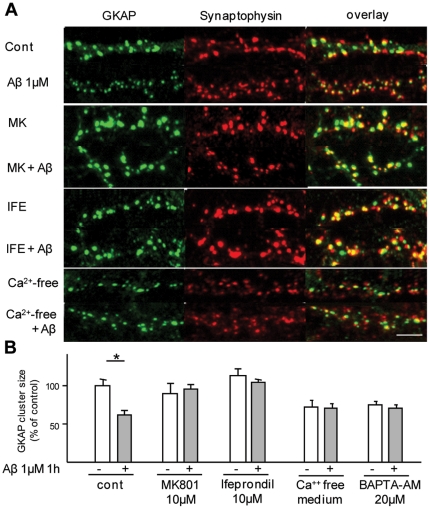
Aβ-induced GKAP downregulation requires NMDAR activity and Ca^2+^ influx. (*A, B*) Rat frontal cortical neurons were pretreated with the non-specific NMDAR blocker MK801 (10 µM), the NR-2B specific blocker Ifenprodil (10 µM) or the Ca^2+^ chelator BAPTA-AM (20 µM); in independent experiments, neurons were exposed to Aβ in Ca^2+^-free medium. Both MK801 (94.9±6.6%, MK+Aβ vs MK alone, p>0.05) and Ifenprodil (93.5±5.5% Ife+Aβ vs Ife alone, p>0.05) prevented GKAP degradation. Likewise, either BAPTA-AM (97.8±7.9% Bapta+Aβ vs Bapta alone p>0.05) or incubation in Ca^2+^-free medium (93.6±7.8, Ca^2+^-free medium+Aβ vs Ca^2+^-free medium alone) prevented Aβ-induced downregulation of GKAP, even though both treatments reduced themselves GKAP cluster size compared to control conditions (71.4±8.3 and 73.6±5.0 for Ca^2+^-free medium and BAPTA, respectively, p>0.05). Scalebar 5 µM.

**Figure 3:**
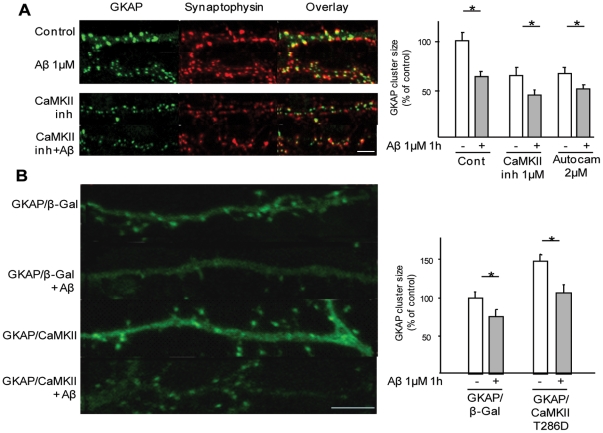
CaMKII regulates GKAP clustering, but does not prevent Aβ-induced GKAP degradation. (*A*) Rat frontal neurons in culture were pretreated with the cell-permeable peptidic inhibitors of CaMKII Autocamtide (2 µM) and CaMKII Inhibitor-I (1 µM) before exposure to Aβ (1 µM, 1 h). Both CaMKII Inhibitor-I and Autocamtide alone decreased GKAP cluster size (64.1±8.3% and 66.5±5.7% of baseline, respectively) but did not prevent a further decrease after Aβ treatment (67.4±8.3% CaMKII inhib+Aβ vs CaMKII alone; 76.4±6.6% Autocamtide+Aβ vs Autocamtide alone). (*B*) CaMKII activation does not prevent Aβ-induced GKAP degradation. Rat frontal neurons were transfected with GKAP-GFP together with either β-gal (negative control) or with the constitutively active mutant of CaMKII (T286D). The mutant CaMKII increased the size of GKAP clusters (142.4±9.6% of β-gal-expressing neurons), but did not prevent the loss of synaptic GKAP after Aβ treatment (72.8±7.0%, CaMKII T286D+Aβ vs CaMKII T286D alone). Scale bar 5 µm.

NMDAR activity regulates synaptic localization and turnover rates of PSD proteins such as PSD95 [13], [28], Homer and Shank [17], [29]. Both, the NMDAR blocker MK801 (10 µM) and the NR2B-specific blocker ifenprodil (10 µM) abolished Aβ-induced GKAP cluster disassembly, indicating the key role of activated NMDAR in the process (Fig. 2A,B); in contrast, blockade of AMPAR or VDCC with NQBX and verapamil, respectively, did not prevent the actions of Aβ (Figure S3A). The role for Ca^2+^ influx in signaling downstream from the NMDAR was proven by the observation that Aβ-induced disassembly of GKAP clusters was abolished under Ca^2+^-free conditions or in the presence of a Ca^2+^ chelator (BAPTA-AM; Fig. 3A,B). On the other hand, neither the src-family inhibitor SU6656 (10 µM) nor the tyrosine phosphatase inhibitor Na_3_VO_4_ (100 µM) influenced GKAP loss (Fig. S3B), ruling out involvement of the tyrosine kinase pathway [30]. Taken together, these data show that an NMDAR-initiated, Ca^2+^-dependent signaling pathway is involved in the control of GKAP downregulation.

### Role of CaMKII in synaptic GKAP clustering, but not downregulation

While diverse Ca^2+^-dependent signaling pathways have been implicated in the regulation of the localization, function and turnover rates of PSD proteins [17], [31], [32], nothing is know about the regulation of GKAP date. A screen of phospho-proteomic databases of purified PSD [33]–[35] yielded 38 phospho-peptide hits related to the GKAP sequence (Table S1). Annotation of these peptides by applying two independent phosphorylation motif prediction algorithms (Scansite: [36]; Phosida: [37]) identified a number of kinases, including CaMKII, several isoforms of PKC, PKA, PKB, CK1 and Cdk5 (Figure S4) that could potentially target GKAP; however, several sites (9 with Phosida, 27 with Scansite, 4 with both algorithms) could not be unambiguously assigned to any individual kinase.

Initial investigations focused on CamKII since this kinase is known to regulate the dynamics of PSD proteins, including SAP97 [38] and PSD-95 [39]. Notably, treatment of neurons with two different peptide inhibitors of CaMKII alone caused a decrease in GKAP cluster size (64.1±8.3% and 66.5±5.7% of baseline in the presence of CamKII inhibitor I and Autocamtide, respectively, Fig. 3A). Further, cotransfection of neurons with plasmids encoding GKAP-GFP and a constitutively active mutant of CaMKII (T286D) increased GKAP cluster sizes (142.4±9.6% of control, GKAP-GFP/β-gal-expressing neurons, Fig. 3B), but did not prevent the effects of Aβ on synaptic GKAP (Fig. 3B). Thus, CaMKII promotes the accumulation of GKAP in the synapse, but plays no role in the downregulation of GKAP by Aβ.

### Cdk5 mediates Aβ-induced GKAP downregulation

**Figure 4:**
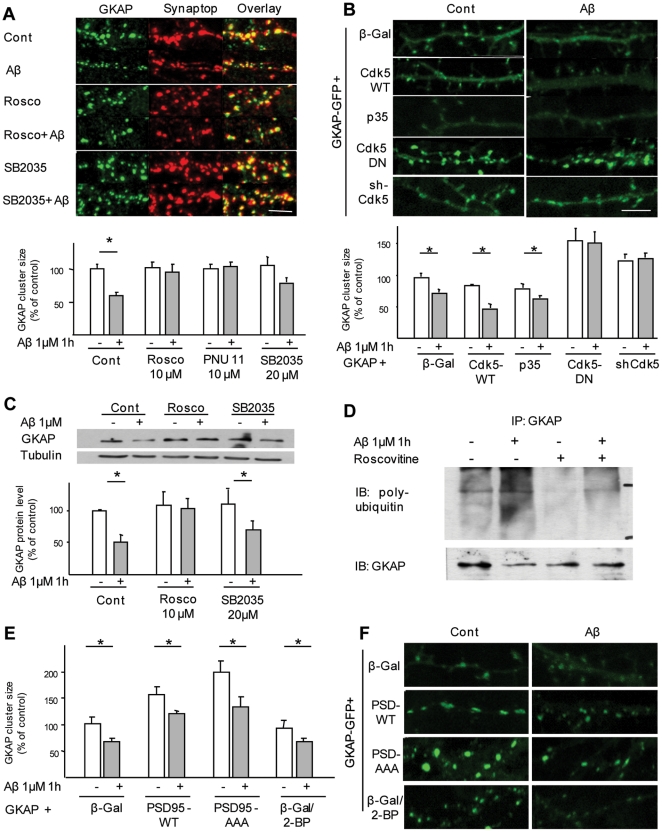
Cdk5 activity is required for Aβ-induced GKAP down-regulation. (*A*) Roscovitine prevents GKAP degradation. Rat-cultured neurons were pretreated with Cdk5 inhibitor Roscovitine (10 µM) or PNU 11, p38 kinase inhibitor (SB2035, 20 µM), before exposure to Aβ. Roscovitine and PNU11 effectively prevented GKAP down-regulation (103.7±11.4% Rosco+Aβ vs Rosco alone; 111.7±8.6% PNU11+Aβ vs PNU alone, both p>0.05) whereas SB2035 partially effective (71.8±7.3 SB+Aβ vs SB alone, p<0.05). (*B*) Expression of cdk5-dominant negative prevents Aβ-induced GKAP degradation. Rat frontal neurons were co-transfected at DIV5 with GKAP-GFP together with β-galactosidase (as negative control), cdk5-WT, p35-WT, cdk5-DN or sh-Cdk5 and were exposed to Aβ (1 µM, 1 h) or vehicle at DIV9. In neurons overexpressing cdk5 or p35 GKAP cluster size was decreased at baseline (87.4±3.5% and 67.5±6.6% vs GKAP+β-Gal respectively, p<0.05) and was further decreased by Aβ (57.1±8.2% and 54.7±3.8% after Aβ in neurons overexpressing cdk5 and p35, respectively, p<0.05). On the other hand, in neurons expressing GKAP together with cdk-dominant negative or sh-cdk5, GKAP were significantly larger than in β-gal expressing neurons (159.4±20.2% and 137.3±9.5% of baseline, respectively, p<0.05) and GKAP degradation was completely prevented (100.0±11.3%, cdk5-DN+Aβ vs cdk5-DN alone; 99.0±2.2% shCdk5+Aβ vs shCdk5 alone, p>0.05). (*C*) Roscovitine prevents GKAP degradation in whole cell protein extract. Rat frontal neurons in culture were pretreated with roscovitine, SB2035 or vehicle before Aβ treatment (1 µM, 1 h). Neurons were lysed and GKAP levels were assessed by western blot. Roscovitine completely prevented GKAP degradation (108.8±20.6%, Rosco+Aβ vs Rosco alone, p>0.05, as compared with 50.8±11.1% vehicle+Aβ vs Aβ alone, p<0.05) whereas SB2035 was infective (49.5±13.6%, SB+Aβ vs SB alone, p<0.05). (*D*) Aβ causes the ubiquitination of GKAP in a cdk-5 dependent manner. Rat frontal cortical neurons were pretreated with MG132, with or without roscovitine, before being exposed to Aβ (1 µM, 1 h). GKAP was immunoprecipitated from whole cell lysate and the blots probed with anti-polyubiquitin antibody. Aβ treatment caused the marked increase in GKAP ubiquitination (lane 2) with an increase in polyubiquitin smear. Roscovitine reduced GKAP ubiqutination in basal conditions (lane 3) and greatly decreased Aβ-induced ubiquitination of GKAP (lane 4). (*E,F*) Aβ-induced GKAP down-regulation is independent of PSD-95 degradation. Rat frontal neurons were transfected with GKAP-GFP-WT together with either β-Galactosidase (β-Gal, negative control) or PSD-95-WT or PSD-95-AAA at DIV5–6 and treated with Aβ (1 µM, 1 h) at DiV9. Overexpression of PSD-95 WT and PSD-AAA alone caused the increase in GKAP-GFP cluster size (158.2±12.3% and 202.4±32.5% of GKAP-GFP/βGal, respectively); Aβ treatment determines a significant decrease in GKAP-GFP cluster size (75.6±5.7% PSD-95WT/GKAP-GFP+Aβ vs PSD-95WT/GKAP-GFP alone; 66.8±9.9% PSD-95-AAA/GKAP-GFP+Aβ vs PSD-95-AAA/GKAP-GFP alone, all p<0.05). In a parallel set of experiments, neurons expressing GKAP-GFP/β-Gal were pretreated for 8 h with 2-bromopalmitate (10 µM) before exposure to Aβ (1 µM, 1 h). Baseline GKAP cluster size was not significantly reduced (94.4±13.4% of untreated controls, p>0.05) and Aβ effect was not prevented (70.1±7.0% 2-BP+Aβ vs 2BP alone, p<0.05). Imaging parameters were kept constant across conditions, scale bar 5 µm.

The involvement of kinase pathways identified by *in silico* analysis as potential regulators of GKAP (see Table S1) was screened using a pharmacological approach. Two cdk5 inhibitors, roscovitine and the structurally unrelated PNU112455, abolished Aβ-induced GKAP degradation (Fig. 4A). In comparison, inhibition of p38 (with SB2035) only partially attenuated the actions of Aβ (71.8±7.3%, SB+Aβ vs SB alone, as compared with 53.7±3.4% in control+Aβ vs control alone, p<0.05; Fig. 4A), and blockade of PI-3K, ERK, JNK, PKC and PKA, with Wortmannin, UO126, AEG3482, Gö6893, and H89, respectively, had no significant effect (Fig. S5A). The activation of cdk5 by Aβ was confirmed by monitoring the levels of cdk5 activator p35 and its cleavage product p25 in whole-cell protein extracts; Aβ treatment upregulated p35 and strongly increased the levels of p25 (whose upregulation is responsible for the disregulated activation of cdk5; Fig. S5B); levels of cdk5 were unchanged.

The role of cdk5 was confirmed by co-transfecting cortical neurons with GKAP-GFP and either cdk-5 or p35, its activating partner. Overexpression of cdk5 or p35 led to a significant decrease in GKAP-GFP cluster size (p<0.05), and significantly potentiated the effects of Aβ (Fig. 4B). Transfection of neurons with *cdk5-DN* or *sh-cdk5* plasmids resulted in GKAP-GFP clusters that were markedly larger than at baseline (Fig. 4B). Notably, Aβ failed to influence GKAP-GFP clusters when cdk5 was depleted using these approaches (Fig. 4B).

Cdk5 was found to have a role that extended beyond the disassembly of GKAP clusters, namely in the regulation of overall GKAP protein levels. Specifically, roscovitine prevented the reduction of GKAP levels in whole cell protein extracts after Aβ application (Fig. 4C). Since Aβ-induced GKAP degradation was proteasome-dependent (Figure S5C), we next tested the involvement of cdk5 in GKAP ubiquitinylation by probing immunoprecipitated GKAP from vehicle- and Aβ-treated cells with a poly-ubiquitin antibody. Cells exposed to Aβ displayed a stronger polyubiquitinylated GKAP signal (Fig. 4D, lanes 1 and 2) whereas roscovitine reduced baseline levels of ubiquitinylated GKAP and markedly attenuated Aβ-induced GKAP ubiquitinylation (Fig. 4D, lanes 3 and 4). Taken together, these findings establish the involvement of cdk5 in Aβ-induced GKAP degradation.

Previously, cdk5 was shown to phosphorylate PSD95, whose degradation after Aβ treatment requires phosphorylation by cdk5 [13], [31]. Therefore, to examine whether Aβ-induced GKAP degradation is directly or indirectly regulated by cdk5, frontocortical neurons were transfected with *GKAP-GFP* together with wild type *PSD95* or a mutated form of PSD95 (PSD-AAA) whose cdk5 phospho-acceptor sites were replaced by alanine residues [31]. Like neurons overexpressing wildtype *PSD-95*, those expressing *PSD-AAA* displayed GKAP clusters that were significantly larger than those found in control (β-Gal-transfected) neurons (Fig. 4E,F). However, application of Aβ led to a comparable decrease in GKAP cluster size in neurons expressing either wildtype *PSD95* or *PSD-AAA* (Fig. 4E,F), indicating that while both constructs increase synaptic recruitment of GKAP, Aβ-induced loss of GKAP occurs independently of PSD-95 phosphorylation. Supporting the view that GKAP degradation can be dissociated from PSD95 degradation, depletion of synaptic PSD95 with 2-bromopalmitate, an inhibitor of palmitoylation [40], did not influence the ability of Aβ to downregulate GKAP (Fig. 4E,F).

### Cdk5 interacts with GKAP and regulates GKAP phosphorylation at specific sites

**Figure 5:**
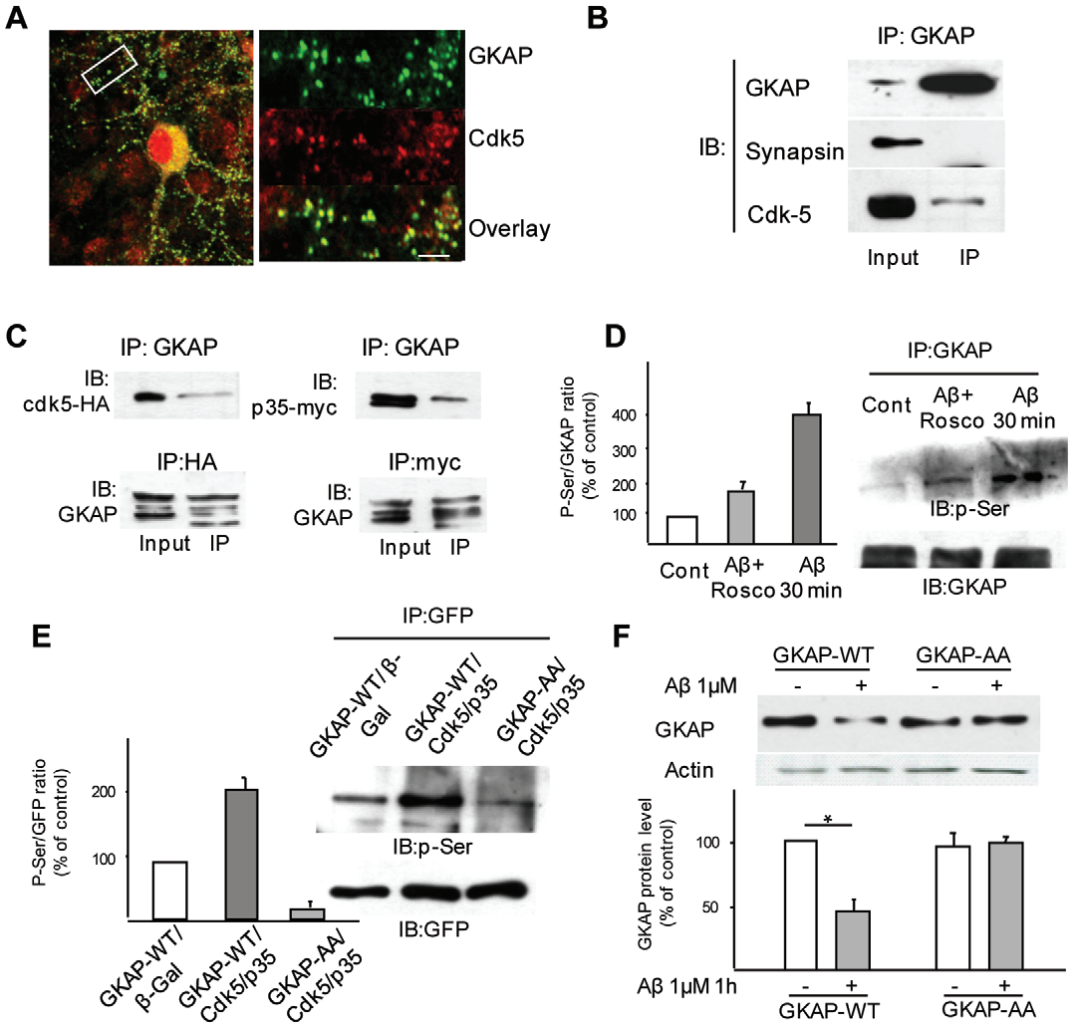
Degradation of GKAP after Aβ treatment is mediated by cdk-5-dependent phosphorylation of two epitopes at S77 and S111. (*A*) GKAP and cdk5 colocalize in synaptic sites. Rat frontal neurons were immunostained for GKAP and cdk5; in dendrites, both proteins displayed a punctuate staining with very high colocalization (95.4% of GKAP-positive puncta were cdk5-positive). (*B*) GKAP immunoprecipitated from cortical synaptosomes was detected with an anti cdk5 antibody (with significant quantity of cdk5 identified); no signal was visualized upon blotting with anti-synapsin antibody. (*C*) SK-N-MC cells were transfected with either GKAP-GFP and p35-HA or with GKAP-GFP and cdk5-myc. Immunoprecipitation of GKAP efficiently co-purified both p35 (detected by anti-HA antibody) and cdk5 (detected by anti-myc antibody); in the reverse order of the experiment immunoprecipitation with anti-HA and anti-myc antisera efficiently enriched for GKAP-GFP. (*D*) GKAP phosphorylation is increased by Aβ in a cdk-5-dependent manner. Rat frontal cortical neurons were treated with Aβ 1 µM for 30 min with or without pretreatment with roscovitine. GKAP was immunoprecipitated and probed with an anti-phosphoserine antibody mix. Aβ treatment caused a marked increase in GKAP phosphorylation (384.2±21.5% of baseline, p<0.05, lanes 1 and 3), but this effect was greatly reduced by roscovitine (173.8±28.1%, lane 2). (*E*) GKAP-S77/111A (GKAP-AA) escapes from cdk5 phosphorylation. SK-N-MC cells were transfected with either GKAP-GFP-WT+βGal (negative control), or with GKAP-GFP-WT+p35+cdk5 or with GKAP-GFP-AA+p35+cdk5/as depicted. GKAP WT or the mutated form were immunoprecipitated with an antibody against GFP and tested with an antibody against phospho-serine. Co-transfection with cdk5/p35 greatly increased the phosphorylation of GKAP-WT compared to baseline (lane 1 and 2), but did not increase the phosphorylation of GKAP-AA (lane 3). (*F*) GKAP-AA resists Aβ-induced degradation. SK-N-MC cells were transfected with either GKAP-WT or GKAP-AA and treated with Aβ (1 µM, 1 h) or vehicle. Aβ caused a decrease of GKAP-WT levels by 46.4±8.8% (p<0.05) of control, whereas GKAP-AA levels were not affected by Aβ (99.8±4.4%, GKAP-AA+Aβ vs GKAP-AA alone, p>0.05).

Since cdk5 plays an important role in the degradation of GKAP, we were prompted to ask whether GKAP is a direct target of this kinase, as suggested by the *in silico* identification of at least two putative phosphorylation sites (Table S1). Cdk5 and GKAP were found to colocalize extensively within synaptic sites of mature neurons (87.4±4.5% of GKAP clusters were cdk-5 positive, Fig. 5A). Moreover, cdk5 and GKAP could be co-immunoprecipitated from Triton X-100-solubilized cortical synaptosomes (Fig. 5B); supporting a transient interaction between cdk5 and its substrate, cdk5 could not be co-immunoprecipitated when a stronger detergent (deoxycholate/NP40) was used. Further evidence for direct interaction between GKAP and cdk5 was provided by expressing GFP-tagged GKAP together with HA-tagged cdk5 or myc-tagged p35 in SK-N-MC neuroblastoma cells. Antibodies directed against the HA or myc epitopes allowed pulling down of GKAP-GFP, and in a reverse experiment, anti-GKAP immunoprecipitates were enriched in cdk-5-HA or p35-Myc (Fig. 5C). We then verified Aβ-induced GKAP phosphorylation in cortical neurons by probing immunoprecipitated GKAP with a phospho-Serine-specific antibody. As shown in Fig. 4D, Aβ treatment markedly increased overall phosphorylation levels of GKAP (lanes 1 and 3), but GKAP phosphorylation was greatly reduced by roscovitine (Fig. 5D).

*In silico* analysis predicted at least two cdk5 consensus sites within the GKAP sequence located in the N-terminus of GKAP (SP motifs at S77 and S111 in the GKAP sequence, corresponding to S403 and S437 in the full-length SAPAP1 isoform, Fig. S4). To establish the role of these two SP motifs as targets of cdk5 we mutated both serines to alanine residues (GKAP S77/111A, hereinafter named GKAP-AA). Since *in vitro* kinase assays may inherently suffer from specificity, we co-expressed *GKAP-GFP-WT* and *GKAP-GFP-AA* together with *cdk5* and *p35* (or *β-Gal* as control) in SK-N-MC cells; GKAP was then immunoprecipitated and a phospho-Serine antibody was used as probe. While overexpression of *cdk5/p35* resulted in markedly increased phosphorylation of GKAP-GFP-WT (further supporting the role of cdk5 in phosphorylating GKAP), the phosphorylation status of the GKAP-AA double mutant was unchanged (Fig. 5E). Furthermore, in SK-N-MC cells (which express endogenous NMDAR, p35 and cdk5 expression, [13]) transfected with GKAP-GFP-WT, treatment with Aβ resulted in a significant decrease in GKAP protein levels in whole cell extract, whereas GKAP-GFP-AA proved resistant to the effects of Aβ (Fig. 5F).

### GKAP-AA mutant is resistant to degradation and prevents Aβ-induced actin remodeling in synapses

**Figure 6:**
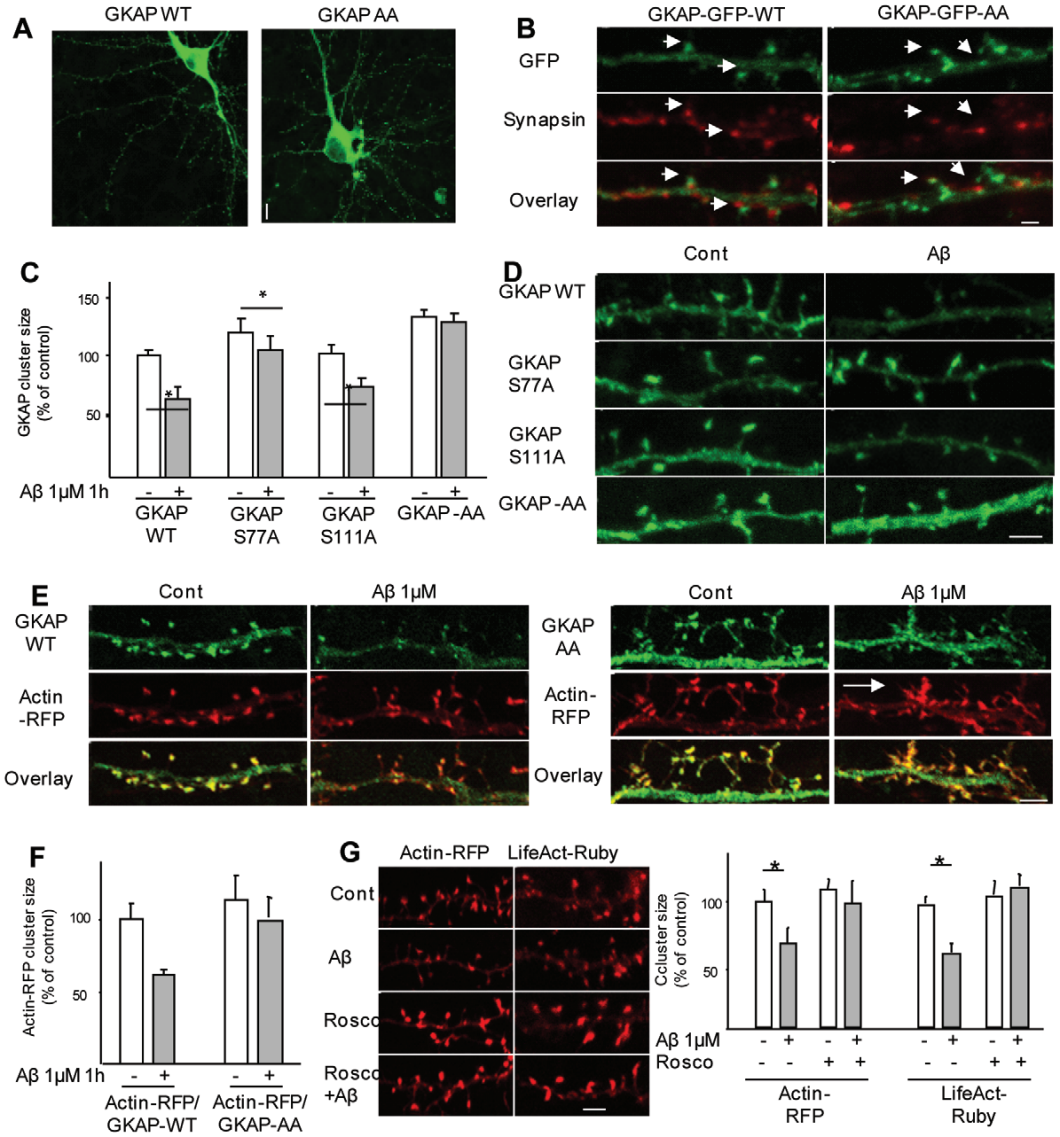
Blockade of GKAP degradation prevents Aβ-induced actin cytoskeleton remodeling. (*A,B*) GKAP-AA is localized in synapses. Rat frontal neurons were transfected with either GKAP-WT or GKAP-AA, fixed and immunostained for synapsin-I. Synapsin-I clusters opposed to the majority of GKAP clusters, confirming the proper trafficking of the mutated form of GKAP. (C,D) GKAP-AA is resistant to Aβ-induced degradation in dendritic spines. Rat frontal cortical neurons were transfected with either GKAP-WT-GFP or with the single point mutation GKAP-S77A-GFP and GKAP-S111A-GFP or with the double point mutation GKAP-AA. Upon Aβ treatment, GKAP-WT cluster size decreased to 64.4±11.5% (p<0.05) of vehicle-treated control. Cluster size for GKAP-S77A-GFP and GKAP-S111A-GFP decreased to 88.6±10.7% and 74.8±6.9% of vehicle-treated, respectively (p<0.05); GKAP-AA clusters were completely resistant to Aβ-induced down-regulation (108.0±9.1% of vehicle-treated, p>0.05). Scale bar 5 µm. (*E,F*) Rat frontal cortical neurons were transfected at DIV5–6 with Actin-RFP together with either GKAP-WT-GFP or GKAP-AA-GFP and treated with Aβ (1 µM, 1 h) at DIV 9–10. In control conditions (*A*), GKAP-GFP and Acti-RFP were highly colocalized and both were enriched in dendritic spines. Upon Aβ treatment, both GKAP-WT-GFP and Actin RFP cluster size decreased (for actin, 61.3±4.5% of control, p<0.05). In GKAP-AA-expressing neurons, actin-RFP cluster size was not affected by Aβ (94.7±13.9%, GKAP-AA+Aβ vs GKAP-AA alone, p>0.05). However, abnormal clusters of GKAP-AA and actin could be seen (panel B, arrow). (*G*) Roscovitine prevents Aβ-induced actin remodeling. Rat cortical neurons were transfected with actin-RFP and pretreated with roscovitine (10 µM) or vehicle before being exposed to Aβ (1 µm, 1 h). In vehicle-pretreated neurons Aβ decreased actin-RFP cluster size by 67.1±13.3% (p<0.05); on the other hand, roscovitine completely blocked Aβ effect (90.9±15.1% of control, p>0.05). Likewise, Aβ caused a marked decrease in F-actin accumulation in spines (61.5±6.7% of control, p<0.05), whereas roscovitine pre-treatment largely blocked this effect (90.0±15.1%, Rosco+Aβ vs Aβ alone). Scale bar 5 µm.

To confirm the role of cdk5 and of the two putative phosphorylation sites in Aβ-induced GKAP degradation, we transfected mature primary neurons with GKAP-GFP-WT or AA. When expressed in cortical neurons, both GKAP-GFP-WT and GKAP-GFP-AA displayed a punctuate distribution along dendrites (Fig. 6A,B) and the clusters of both WT and mutant protein colocalized with synaptophysin puncta (Fig. 6B). Notably, cluster size distribution of GKAP-GFP-AA was significantly rightward shifted (i.e. toward larger clusters) compared with the WT protein (not shown). Application of Aβ to cortical neurons that had been transiently transfected with either *GKAP-GFP*-WT, double-mutated *GKAP-AA* or the single-mutated *GKAP-S77A* or *GKAP-S111A*, resulted in a major decrease in GKAP cluster size in *GKAP-WT*-expressing cells (64.4±11.5% of control, p<0.05), whereas the changes in GKAP levels were smaller in cells expressing the S77A (88.6±10.7%) and S111A (74.8±6.9%) mutations and absent in cells transfected with *GKAP-AA* (108.0±9.1%) (Fig. 6C,D). Taken together, these sets of data suggest that Aβ triggers the phosphorylation of GKAP by cdk5 at the serine residues S77 and S111 that in turn are crucial for GKAP degradation.

Previous work identified the remodeling of actin structures among the synaptic effects of Aβ [10]. Since GKAP and other SAPAP family members interact with actin-regulating proteins such as nArgBP2, Shank and pyk2 [25], [41], [42] we investigated whether GKAP loss impacted on Aβ-induced modifications of the actin cytoskeleton. Under baseline conditions, tranfected cortical neurons displayed extensive colocalization of actin-RFP with exogenously expressed GKAP-GFP-WT and GKAP-GFP-AA (Fig. 6E,F). Treatment of neurons with Aβ resulted in marked reductions in GKAP-WT-GFP (54.5±11.3%, p<0.05) and actin-RFP (61.3±4.5% of control, Fig. 6E,F) cluster sizes. When the same treatment was applied to neurons transfected with GKAP-AA-GFP, no marked changes in either GKAP-AA-GFP (92.3±14.6, p<0.05; Fig. 6E,F) or actin-RFP (94.7±13.9% of control, p>0.05, Fig. 6E,F) cluster sizes were observed. Notably, GKAP-GFP-AA-expressing neurons displayed an increase in elongated spines with split head and sporadically abnormally large actin clusters colocalized with GKAP-GFP-AA clusters were detected.

Thus, preventing cdk-5 phosphorylation of GKAP (by expressing the GKAP-AA mutant) prevents Aβ effect on actin cytoskeleton. To confirm the involvement of cdk5 in Aβ-induced actin remodeling, cortical neurons were transfected with actin-RFP (or with the F-actin sensor Lifeact-Ruby, [43]) and pretreated with roscovitine before exposure to Aβ (1 µM, 1 h). Whereas Aβ markedly decreased actin cluster size (67.1±13.3% of baseline, Fig. 6G), roscovitine pretreatment largely abolished this effect (90.9±15.1% of control; Fig. 6G). These findings were confirmed in neurons expressing the F-actin sensor Lifeact Ruby (Fig. 6G).

## Discussion

The principal findings of the present work are that exposure to Aβ results in the removal and degradation of synaptic GKAP, the latter being a consequence of cdk5 activity and phosphorylation of two consensus sites in the N-terminus of the protein.

The AD brain accumulates the 40 and 42 amino acid forms of Aβ peptide, the latter being the principal component of amyloid fibrils and plaques [44]. In contrast, Aβ40 peptide is highly represented in the soluble amyloid fraction [44] and its levels correlate with synaptic loss in pathological specimens from AD patients [44], [45]. Imprtantly, while Aβ42 is prone to form aggregates, Aβ40 stably assembles into low-n oligomers [46] and is considered responsible for memory and synaptic dysfunction [47], [48]. Given these facts, the Aβ40 peptide was used in these studies to study Aβ-associated neurotoxiciry without the confounding effects of high-molecular weight aggregates.

Aβ peptides exert a profound effect on synaptic structures [11], [49], [50]. Prominent among these effects are the degradation and loss of a number of PSD proteins [7], [10], [13], effects that lead to the collapse of synapses. PSD-95 is a major constituent of the PSD, participating in the trafficking and signaling of glutamatergic receptors [16]. Levels of PSD-95 are down-regulated by Aβ, both *in vivo* [45] and *in vitro* [13]), and the loss of PSD-95 correlates with the removal of AMPA receptors [13]. Aβ-induced degradation of PSD95 is dependent upon NMDAR activity and phosphorylation by cdk5 at three serine residues located in the N-terminal domain, whose replacement by alanine protects PSD-95 from Aβ-induced proteasomal degradation [13], [31]. The results of the present study show that GKAP is also regulated by cdk5 after NMDAR activation.

The involvement of NMDAR in Aβ-induced disassembly of the PSD adds to the growing spectrum of effects of Aβ that depend on NMDAR activity. Previous studies have shown that the NMDA glutamate receptor is required for synaptic targeting of Aβ oligomers [51] and that NMDAR activity is required for Aβ-induced blockade of axonal transport and triggering of oxidative stress as well as mitochondrial fragmentation and changes in spine morphology [52]–[54]. These findings suggest that NMDAR constitute the first hub in Aβ signaling cascades.

The present work identifies cdk5 as a major downstream cascade of NMDAR signalling. Moroever, it establishes GKAP as bona fide cdk5 substrate since i) cdk5 and GKAP interact physically; ii) overexpression of cdk5 increases GKAP phosphorylation; iii) blockade of cdk5 substantially reduces GKAP phosphorylation; and iv) mutation of the cdk5 consensus sites on GKAP prevents GKAP phosphorylation. As in the case of PSD-95, Aβ-induced phosphorylation of GKAP leads to proteasomal degradation, and phosphorylation-resistant mutants of both proteins fail to undergo degradation after Aβ treatment. Importantly, however, the final steps of their degradation diverge beyond cdk5: PSD-95 is ubiquitinated by mdm-2 [28], whereas GKAP becomes a substrate for TRIM3 [55]. Further, GKAP degradation occurs even in neurons expressing a degradation-mutant of PSD-95 (Fig. 3E,F), indicating that GKAP and PSD-95 can occur independently of each other. Since a number of membrane-associated guanylate kinase (MAGUK) scaffold proteins such as PSD-93, PSD-95, SAP102 and CASK [31], [56] carry N-terminal cdk5 consensus sites, it appears that cdk5 is part of a regulatory pathway common to MAGUKs (and, potentially, to other non-GUK proteins, e.g. SPAR; [57]). The wide representation of cdk5 consensus sites in scaffold proteins suggests that cdk5 has a ‘gatekeeper’ role in PSD assembly/disassembly, notwithstanding minor contributions by other kinases (e.g. CaMKII, see [39]; potentially p38k, see [58]). The findings reported here bolster the view that cdk5 plays a critical role in AD; previous work showed its importance in mediating Aβ-induced synaptic dysfunction [59] and PSD protein degradation [13], [17] as well as in tau hyperphosphorylation, transcriptional derangement, amyloidogenesis and neuronal apoptosis [60]–[63]. While cdk5 appears to be central in Aβ-induced degradation of PSD-95 and GKAP [13], [17 and results presented here], other Aβ effects, such as NMDAR and Insulin receptors [64], are dependent upon CaMKII activity. However, our experiments reveal that CaMKII is not involved in GKAP degradation. Taken together, these findings underscore the divergence of signalling cascades that are initiated by Aβ and the segregation of their synaptic effects.

Derangement of the actin cytoskeleton of synapses is likely to be a crucial step in Aβ-triggered synapse demise [11], [10]; to date, at least one pathway involving LIM kinase inactivation, calcineurin activation and dephosphorylation of the actin-depolymerizing factor cofillin has been implicated in this process [11], [15]. However, since GKAP interacts with several proteins involved in actin regulation (e.g. nArgBP2, Shank and pyk2; [19], [25], [41]) and since blockade of GKAP degradation results in increased dendritic spine size [55], it is plausible that GKAP loss may be, at least partly, responsible for the actin derangement observed after Aβ treatment [10]. Supporting this notion, we found that expression of phosphorylation-resistant GKAP or treatment with cdk-5 inhibitors counteracts Aβ-induced actin depolymerization. The finding that GKAP degradation contributes to the collapse of actin structures [10], strengthens the view that GKAP is important for maintaining the organization of the actin cytoskeleton in dendritic spines. In fact, cdk5 and its activators can associate with actin structures [65], [66] and several actin-regulating proteins (e.g. WAVE, ephexin-1 and spinophillin) are known cdk5 substrates [67]–[69]. Moreover, cdk5 has been shown to act as a negative regulator of the actin cytoskeleton, and phospho-WAVE and -ephexin-1 prevent actin polymerization [68], [69], induce the collapse of actin structures, and cause dendritic spine retraction [70]. Extrapolating from these various findings, it would appear that cdk-5-mediated GKAP degradation disconnects the actin structures from the PSD and/or influence the localization of actin-regulatory proteins; in the context of the present work, GKAP loss is likely to occupy a central position in the cascade leading from PSD disassembly to Aβ-triggered collapse of the synaptic actin cytoskeleton.

## Materials and Methods

### Ethic Statement

The study was approved by the Government of Upper Bavaria (Regierung von Oberbayern, Muenchen) Animal welfare committee (authorization number 55.2-1-54-2531-169-09) and conformed international (EU Directive 86/609) standards on animal welfare and experimentation. All possible steps were taken toward reducing the number of animals used and their suffering.

### Drugs and chemicals

Aβ_1–40_ (American Peptides, Sunnyvale, CA) was prepared as previously described [13] to yield predominantly low N-oligomers (mainly monomeric to tri-tetrameric, see Fig. S1) [71]–[73] rather than Aβ fibrils [74], [75]. Aβ1–42 (Bachem, Bubendorf, CH) was resuspended in Hexafluoro-2-propanol (HFIP)and soluble oligomers were prepared according to [64]. Briefly, the peptide was dissolved in HFIP to 1 mM and stored as a dried film at −80°C after solvent evaporation. The film was resuspended in DMSO to a final concentration of 5 mM, thoroughly vortexed and sonicated for 10 min. The solution was then diluted with ice-cold neurobasal medium to 100 µM and stored at 4°C (16 h). The solution was centrifuged at 14,000 *g* (10 min) and the supernatant was used for treatment.

Roscovitine, (±)-verapamil, BAPTA-AM, 2-bromo palmitate (2-BP) and cycloheximide were purchased from Sigma Chemicals (Deisenhofen, Germany); MK801 (5S,10R)-(+)-5-Methyl-10,11-dihydro-5H-dibenzo[a,d]cycloheptene-5,10-imine maleate, ifenprodil (1R*,2S*)-erythro-2-(4-Benzylpiperidino)-1-(4-hydroxyphenyl)-1-propanol hemitartrate, NBQX (2,3-Dioxo-6-nitro-1,2,3,4-tetrahydrobenzo[*f*]quinoxaline-7-sulfonamide), UO126 (1,4-Diamino-2,3-dicyano-1,4-bis[2-aminophenylthio]butadiene), wortmannin, lactacystin, AEG3482 (6-Phenylimidazo[2,1-b]-1,3,4-thiadiazole-2-sulfonamide, inhibitor of JNK signaling) and SB2035 (fluorophenyl)-2-[4-(methylsulfonyl)phenyl]-1H-imidazol-4-yl]pyridine, p38 kinase inhibitor) were from Tocris (Bristol, UK); 6-Aminopyridine-sulfonamide (PNU112455A; cdk2/5 inhibitor), sodium orthovanadate, SU6656 (2,3-dihydro-N,N-dimethyl-2-oxo-3-[(4,5,6,7-tetrahydro-1H-indol-2-yl)methylene]-1H-indole-5-sulfonamide) and MG132 (z-Leu-Leu-Leu-al proteasome inhibitor) were from Calbiochem (La Jolla, CA).

### Antibodies

Rabbit polyclonal anti-GKAP antibody was kindly provided by M. Sheng (Cambridge, MA) or purchased (Cat. No. H81) from Santa Cruz Biotechnology (Heidelberg, Germany); anti-myc and anti-HA antisera were also from Santa Cruz. Mouse monoclonal anti-synaptophysin was purchased from Sigma, rabbit polyclonal anti-synapsin I from Chemicon (Temecula, CA), anti-p35, -cdk5, from Cell Signalling (Frankfurt am Main, Germany), anti-phospho-serine mix from Qiagen (Hilden, Germany), anti-polyubiquitin from Beckton Dickinson (Erembodegem, Belgium), and mouse monoclonal anti-GFP from NeuroMab (Davis, CA).

### Primary neuronal cultures

Trypsin-dissociated primary cell cultures were prepared from frontal cortical tissue from 4-day-old (P4) Wistar rats (Charles River, Sulzfeld, Germany), as described previously [17]. Cells were plated onto gelatin/PDL-coated 6-well plates at a density of 450–500 cells/mm^2^ or on glass coverslips (450 cells/mm^2^ for immunostaining and -blotting experiments; 800 cells/mm^2^ for transfection studies). Experiments were started after 10–13 days *in vitro* (DIV 10–13). Studies under Ca^2+^-free conditions were performed by incubating cells in artificial cerebrospinal fluid (NaCl 124 mM, KCl 3 mM, NaHCO_3_ 26 mM, MgSO_4_ 1 mM, KH_2_PO_4_ 1.25 mM, glucose 10 mM) for 30 min before treatment. Likewise, drugs were added to the culture medium 30 min before Aβ treatment.

### Culture of SK-N-MC cell line

Human neuroblastoma SK-N-MC (obtained from ATCC, Cat. No. HTB-10) cells were maintained at 37°C (5% CO_2_/95% air) in DMEM supplemented with 10% FCS (Invitrogen, Darmstadt, Germany) and 1% kanamycin. Cells were seeded on 100 mm Petri dishes (2×10^6^ cells) or 6-well plates (10^5^ cells) 24 h before use.

### Brain fractionation and synaptosome preparation

Synaptosomes were prepared using a previously described method [76]. Briefly, cerebral cortices were dissected out on ice, homogenized in 10 volumes of ice-cold buffer (sucrose 0.32 M and HEPES 5 mM) containing protease (Complete Protease Inhibitor; Roche, Mannheim, Germany) and phosphatase (Phosphatase Inhibitor Cocktails I and II; Sigma) inhibitor cocktails, before centrifugation (1000 *g*, 5 min). The resulting supernatant was centrifuged at 15000 *g* (15 min); the resulting pellet re-suspended in sucrose buffer (0.3 M) and centrifuged (15000 *g*, 15 min.) to yield a crude synaptosomal preparation.

### Plasmids and targeted mutagenesis

The GW1-CMV-GKAP-GFP construct was a gift of C. Sala (Milan, Italy). Constructs encoding p35-myc, cdk5-HA and cdk5-DN-HA were obtained from Addgene (Cambridge, MA; plasmids 1347, 1872, and 1873, respectively). An RNAi against cdk5 in pSilencer2.0 was kindly provided by L.H. Tsai (Cambridge, MA; see [56]). Wild-type PSD-95 and triple alanine (T19A, S25A, S35A) point-mutated PSD-95 were kindly provided by M. Morabito (Worcester, MA) and were previously described [31]. The constructs encoding farnesylated-EGFP and constitutively active CaMKII (T286D) were gifts from M. Zagrebelsky (Braunschweig, Germany) and G. Turrigiano (Waltham, MA), respectively. Plasmids encoding actin-RFP and Lifeact-Ruby were provided by D. Refojo (Munich, Germany) and R. Wedlich-Soldner (Martinsried, Germany).

Site-directed mutagenesis of *GKAP* was performed according to [77] with slight modifications. PCR products were purified on PCR clean-up spin columns (Macherey-Nagel, Dueren, Germany) and the DNA was digested with DpnI (20 U) for 90 min at 37°C, after which Dpn1 was inactivated (65°C, 10 min). DNA was extracted (phenol/chloroform), precipitated and inoculated into transformed, electro-competent bacteria (Invitrogen). Site-directed mutations were confirmed by sequencing.

### Transfection

Neuronal cultures were transfected with a total of 1–1.2 µg DNA per well, using Lipofectamine 2000, following the manufacturer's protocol (Invitrogen). Neurons were transfected at DIV 5–6 and used at DIV 9–11. SK-N-MC cells (70% confluence) were transfected using Jet-PEI (Polytransfection, Illkirch, France) following a previously published protocol [78], using 6 µg or 1–1.2 µg DNA, respectively, when 100 mm plates or 6-well dishes were used. SK-N-MC cells were replenished with fresh medium 16 h after transfection and harvested after 24 h.

### Immunocytochemistry and image analysis

For immunolocalization experiments, neurons grown on coated glass coverslips were fixed in cold methanol (−20°C, 20 min), blocked (5% BSA and 0.001% Triton X-100 in PBS) and incubated with anti-GKAP (1∶1000), anti-synaptophysin (1∶500) or anti- cdk-5 (1∶500) at 4°C (18 h). Following extensive washing, coverslips were incubated (1 h, RT) with secondary anti-mouse (conjugated with Alexa-594, Invitrogen) or anti-rabbit (conjugated with Alexa-488, Invitrogen) before washing and mounting. Transfected neurons were fixed in freshly prepared 4% PFA in PBS (4 min, RT) before mounting. Images were captured with an Olympus FluoView1000 confocal microscope using a plan-apochromat 63×/1.2 water lens, at a resolution of 1024×1024. GKAP cluster size was analyzed using ImageJ software after thresholding at the arbitrary value of 150. Only GKAP clusters justaxposed to synaptophysin puncta (manually selected by an investigator who was blind to the treatments) were evaluated; surface areas were measured with ImageJ software and logged into an Excel file. Clusters separated by 1 pixel were considered to represent individual clusters. To reduce noise effects, only clusters ≥3 pixels were included in the analysis; while this criterion possibly introduced a bias toward smaller differences in treated *vs.* untreated groups in cases where treatment caused a shrinkage of a substantial proportion of clusters to <3 pixels, it would rather lead to an overestimation of average cluster size in the treated groups without undermining the statistical significance of detected differences. The cluster size analysis was complemented by independent evaluation of the images by a second investigator (also blind to treatments) who ranked images according to cluster size; in all cases, there was a 100% match between these latter qualitative evaluations and the quantitative analysis. Co-localization was defined as the number of puncta displaying immunopositive synaptophysin and GKAP puncta/total number of synaptophysin-positive puncta. Only puncta ≥3 pixels were considered to be clusters and included in the analysis. The same procedure was adopted for GKAP-GFP clusters in transfected neurons, except that the arbitrary threshold was set at 210. For estimating the spine/shaft intensity ratio, a fixed-size circular region of interest was placed on the spine head on the underlying dendrite (and in the nearby empty space for background measurements which was then subtracted).

### Electron microscopy

Primary neurons were grown on Permanox slides (LabTek, Nalgene, Naperville IL) at 600 cells/mm^2^ and used after 13 DIV. After treatment, cell monolayers were washed in PBS, fixed for 30 min. (25°C) in 3% PFA/3% glutaraldehyde/0.1 M cacodylate buffer (Electron Microscopy Sciences, Hatfield, PA), post-fixed (2% osmium tetroxide), dehydrated, embedded in Epon resin (Electron Microscopy Sciences), and ultrathin sections (70 nm) were prepared and collected on copper grids. Sections were stained with 0.5% uranyl acetate and 3% lead citrate, and grids were examined with an EM10 Zeiss electron microscope (acceleration voltage: 60 kV). Images were acquired at 50,000× original magnification and at resolution of 1376×1036 pixels. Only artifact-free synapses, with clearly identifiable presynaptic terminals, synaptic clefts, postsynaptic membranes and PSD were selected for analysis [79]. PSD density profiles (4–5 averaged profiles for each PSD) were measured with ImageJ software.

### Immunoprecipitation

Transfected SK-N-MC cells were lysed in modified RIPA buffer (50 mM Tris-HCl, 120 mM NaCl, 1% NP-40, 0.5% deoxycholate, 5 mM MgCl_2_, 1 mM EDTA, plus protease and phosphatase inhibitors); lysates were cleared by centrifugation (12000 *g*, 10 min), and 500 µg solubilized protein aliquots were incubated (18 h) with anti-HA, anti-c-myc or anti-SAPAP1 (1 µg each). Immunocomplexes were precipitated by incubation with 30 µl Dynabeads (Invitrogen) for 1 h at 4°C, washed (2 times) in high-salt buffer (50 mM Tris HCl, 500 mM NaCl, 0.1% NP-40, 0.05% deoxycholate), and eluted in Laemmli buffer. Washed synaptosomal fractions were re-dissolved in either modified RIPA buffer (see above) or Triton X-100 buffer (10 mM Tris-HCl, 2% Triton X-100, plus proteases and phosphatases inhibitors [56]. Triton-solubilized fractions were cleared by centrifugation (12000 *g*, 10 min) and solubilized protein was incubated (18 h) with 2 µg anti-GKAP. Immunocomplexes were precipitated by incubation with protein G/protein A-Agarose mix (Invitrogen) and washed twice with high-salt Triton buffer (100 mM NaCl, 0.2% Triton X-100) before elution in Laemmli buffer.

### Immunoblotting

Neurons were lysed by brief sonication in complete RIPA buffer (50 mM Tris-HCl, 150 mM NaCl, 1 mM EDTA, 5 mM MgCl_2_, 1% NP-40, 0.1% SDS, 0.5% sodium deoxycholate, plus protease phosphatase inhibitor cocktails), before clearing by centrifugation (12000 *g*, 10 min). Cleared lysates (40 µg) were fractionated by electrophoresis on 8% SDS-polyacrylamide (PAGE) gels and transferred onto nitrocellulose membranes which were then blocked (5% nonfat dryed milk powder and 0.2% Tween-20 in PBS) and incubated with the following antibodies: anti-GKAP (1∶1000), anti-synapsin I (1∶1000), anti-GFP (1∶2000), and anti-actin (1∶10,000) or anti-β-tubulin (1∶2000; Oncogene Sciences, Uniondale, NY). Antigens were detected by enhanced chemiluminescence (GE Healthcare, Freiburg, Germany) after incubation with appropriate horseradish peroxidase-IgG conjugates (GE Healthcare); a mixture of anti-mouse IgG and anti-mouse IgM was used in combination with primary anti-phospho-serine antibody.

For amyloid oligomer detections, amyloid peptides were diluted at 1 µM in Neurobasal medium, incubated for 1 h at 37°, loaded in non/denaturing Tricine PAGE buffer (Tris 200 mM, 40% Glycerol) and fractionated on a 4–15% acrylamide gradient in 100 mM Tris/100 mM Tricine/0.1% SDS buffer. The gel was transferred on nitrocellulose membrane, blocked and incubated with antibody against amyloid-β (1∶1000, Cell Signaling). After incubation with HRP-conjugated secondary antibody, the peptide bands were revealed using SuperSignal Femto substrate (Pierce).

Blots were scanned and quantified using TINA 3.0 bioimaging software (Raytest, Straubenhardt, Germany). Linearity was routinely checked during semi-quantification of all blots. All values were normalized and expressed as percentages of controls; in pharmacological experiments, percentages were calculated as Aβ-treated vs Aβ-untreated. Each set of depicted numerical data was obtained from 3–5 independent sets of experiments, with 3 replicates in each run.

### Statistics

All data are depicted as mean ± SD (3–5 independent experiments). Immunofluorescence data derive from evaluation of a minimum of 600 synapses (N) in each of 8–10 neurons (n). Data were analyzed for statistical significance using ANOVA and appropriate *post hoc* tests (Student-Keuls or Kruskal-Wallis multiple comparison procedures, as appropriate) where *p*<0.05 was set as the minimum level of significance.

## Supporting Information

Figure S1 **Amyloid-β forms low-N oligomers in culture medium.** Amyloid-β 40 was diluted in the culture medium at the concentration used to treat neurons and incubated at 37°C for 1 h. Samples of incubated, diluted Aβ were resolved on polyacrylamide gradient gel under non-denaturating conditions and probed with anti Aβ antibody. Three prominent bands were detected, in the range between 4 and 12 kDa, corresponding to monomers to trimers of the peptide.
(TIF)

Figure S2 **Aβ effect on GKAP cluster size is dose-dependent.** Rat cortical neurons were treated with different concentrations of Aβ for 1 h before fixation and immunostaining; GKAP cluster size was not affected by 10 nM Aβ (103.3±9.0% of baseline), but was dose-dependently reduced by treatment with 100 nM, 1 µM, 10 µM Aβ for 1 h (85.3±6.3%, 64.9±4.0% and 57.1±1.2%, respectively, p<0.05).
(TIF)

Figure S3 **AMPAR, VDCC and tyrosine kinases are not involved in GKAP down-regulation.** (*A*) AMPAR and VDCC are not involved in Aβ-induced degradation of GKAP. Pretreatment of rat cortical neurons with either the AMPAR blocker NQBX (25 µM) or the VDCC blocker Verapamil (50 µM) before exposure to Aβ (1 µM, 1 h) did not prevent GKAP down-regulation in synaptic clusters (53.8±6.3% and 54.4±6.0, respectively, p<0.05). (*B*) Tyrosine kinases are not required for Aβ-induced down-regulation of GKAP. Rat cultured neurons were pretreated with or with the src-family inhibitor SU6656 (10 µM) or with the tyrosine phosphatase inhibitor Na_3_VO_4_ (100 µM) before treatment with Aβ (1 µM,1 h); neither compound prevented Aβ effects (63.7±8.5, SU+Aβ vs SU alone, p>0.05; 70.9±14.5, Na_3_VO_4_+Aβ vs Na_3_VO_4_ alone, p>0.05).
(TIF)

Figure S4 **Putative phosphorylation sites targeted by cdk5 and CamKII.** Phosphopeptide database (see Table S1) was interrogated using the GKAP sequence; the resulting GKAP phosphoepitopes were annotated using two kinase-target prediction software (Scansite and Phosida).
(TIF)

Figure S5 **Aβ-induced GKAP degradation does not require PI-3K, ERK, Jnk and PKC activity, but depends on proteasome activity.** (*A*) Rat frontal neurons were pretreated with PI-3K inhibitor Wortmannin (2 µM), ERK inhibitor UO126 (10 µM), Jnk inhibitor (AEG 100 µM) or PKC inhibitor Gö6893 (5 µM) before being exposed to Aβ (1 µM, 1 h). All inhibitors were ineffective (51.5±8.1 Wort+Aβ vs Wort alone, p<0.05; 59.6±9.2 UO+Aβ vs UO alone, p<0.05; 61.7±7.2 AEG+Aβ vs AEG alone, p<0.05; 67.3±5.5 Gö+Aβ vs Gö alone, p<0.05). (B) Rat frontal cortical neurons were treated with Aβ (1 µM) for 0.5, 1, 3, 6 or 24 h. The levels of cdk5 and of the cdk5 activator p35 and the truncated p25 were assessed in whole-cell lysate. Cdk5 levels were not significantly affected by Aβ (111.3±11.4%, 122.2±33.0%, 110.4±23.1%, 109.2±32.3%, 109.4±9.4% at 0.5, 1, 3, 6, and 24 h timepoints, respectively; p>0.05), whereas p35 levels were significantly increased, peaking at 3 h (135.2±16.0%, 216.4±18.9%, 247.8±49%, 176.3±7.2%, 0.86±0.42% at 0.5, 1, 3, 6, and 24 h timepoints) (*C*) Aβ-induced GKAP degradation requires proteasomal activity. Rat prefrontal neurons were pretreated with the proteasome inhibitors MG132 (1 µM) or lactacysthine (10 µM) or the lysosome pathway blocker chloroquine (100 µM) before being exposed to Aβ (1 µM, 1 h). Aβ effect on GKAP cluster size was completely prevented by MG132 and lactacysthine treatment (107.5±8.3% MG+Aβ vs MG alone, p>0.05; 109.3±7.4%; lacta+Ab vs lacta alone, p>0.05) whereas chloroquine was ineffective (65.4±11.1%, chloroquine+Aβ vs Aβ alone, p<0.05).
(TIF)

Table S1 **In silico analysis identifies kinase candidates for the regulation of GKAP.** We interrogated a database containing proteomic data regarding post-translational modifications of synaptic proteins (available at http://www.phosphosite.org/) using the reference sequence of SAPAP1 as query, 38 phospho-peptides were retrieved. The phosphopeptides were annotated using Scansite and Phosida phosphorylation site prediction algorithm. Scansite assigned 16 out of 38 phospho-sites to specific kinases; Phosida identified 28 out of 38 potential kinase-phosphosite relationships. Kinases potentially able to phosphorylate GKAP included cdk5, CaMKII, GSK3, PKA, PKC, Akt, and CK. Seven sites were independently confirmed by the two algorithms as potential substrates of cdk5 (according to SAPAP1 reference sequence S134, S403, S437), CaMKII (S666), PKA (S381). For 5 peptides, no annotation was provided by any software.
(DOCX)
