# Supplementary figures and images for: CDK5 Is Essential for Soluble Amyloid β-Induced Degradation of GKAP and Remodeling of the Synaptic Actin Cytoskeleton
Source: PLoS One. 2011 Jul 29;6(7):e23097. doi: 10.1371/journal.pone.0023097 (PMC3146526; doi:10.1371/journal.pone.0023097)

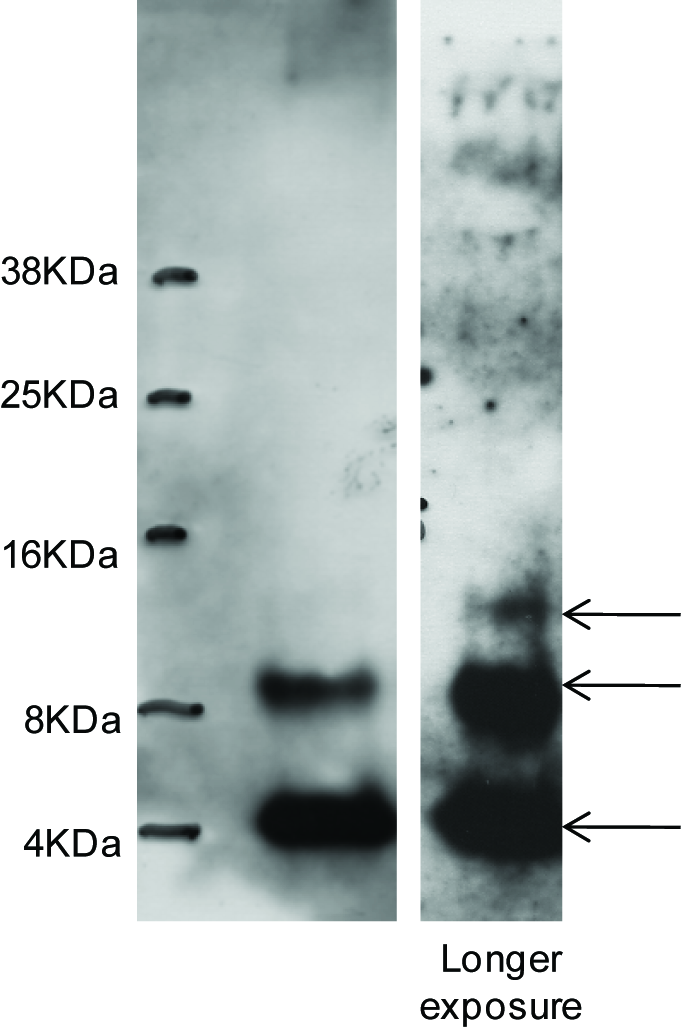

Supplement: Figure S1 — Amyloid-β forms low-N oligomers in culture medium. Amyloid-β 40 was diluted in the culture medium at the concentration used to treat neurons and incubated at 37°C for 1 h. Samples of incubated, diluted Aβ were resolved on polyacrylamide gradient gel under non-denaturating conditions and probed with anti Aβ antibody. Three prominent bands were detected, in the range between 4 and 12 kDa, corresponding to monomers to trimers of the peptide. (TIF) [file pone.0023097.s001.tif]

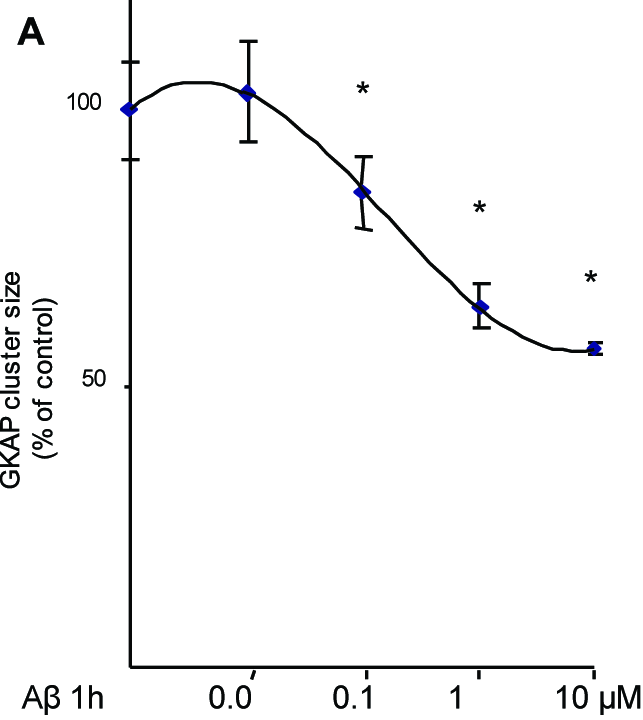

Supplement: Figure S2 — Aβ effect on GKAP cluster size is dose-dependent. Rat cortical neurons were treated with different concentrations of Aβ for 1 h before fixation and immunostaining; GKAP cluster size was not affected by 10 nM Aβ (103.3±9.0% of baseline), but was dose-dependently reduced by treatment with 100 nM, 1 µM, 10 µM Aβ for 1 h (85.3±6.3%, 64.9±4.0% and 57.1±1.2%, respectively, p<0.05). (TIF) [file pone.0023097.s002.tif]

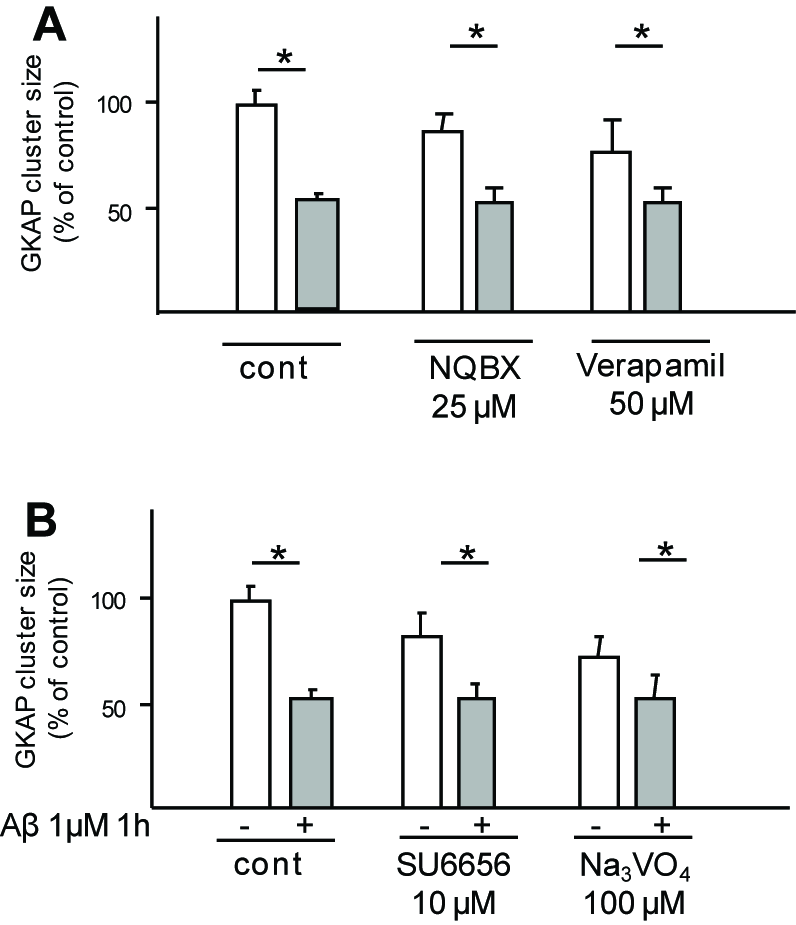

Supplement: Figure S3 — AMPAR, VDCC and tyrosine kinases are not involved in GKAP down-regulation. (A) AMPAR and VDCC are not involved in Aβ-induced degradation of GKAP. Pretreatment of rat cortical neurons with either the AMPAR blocker NQBX (25 µM) or the VDCC blocker Verapamil (50 µM) before exposure to Aβ (1 µM, 1 h) did not prevent GKAP down-regulation in synaptic clusters (53.8±6.3% and 54.4±6.0, respectively, p<0.05). (B) Tyrosine kinases are not required for Aβ-induced down-regulation of GKAP. Rat cultured neurons were pretreated with or with the src-family inhibitor SU6656 (10 µM) or with the tyrosine phosphatase inhibitor Na3VO4 (100 µM) before treatment with Aβ (1 µM,1 h); neither compound prevented Aβ effects (63.7±8.5, SU+Aβ vs SU alone, p>0.05; 70.9±14.5, Na3VO4+Aβ vs Na3VO4 alone, p>0.05). (TIF) [file pone.0023097.s003.tif]

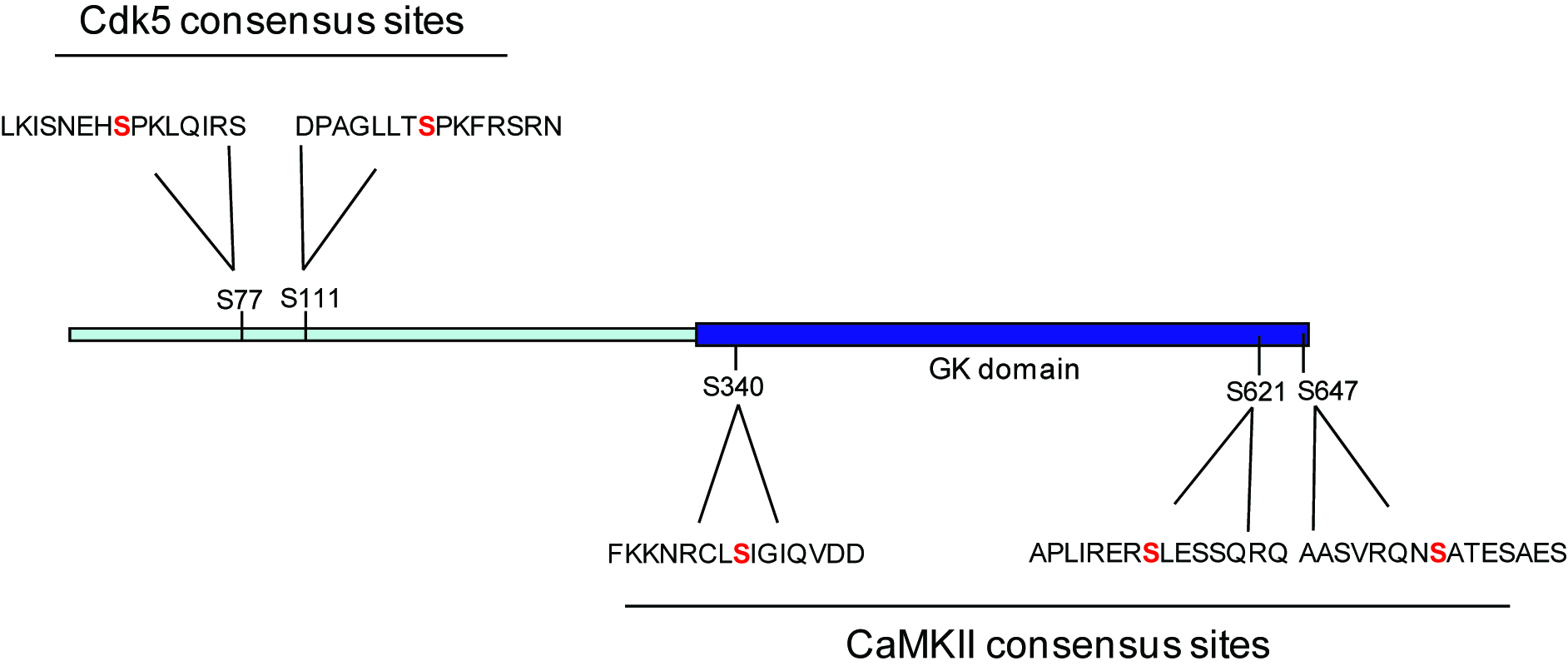

Supplement: Figure S4 — Putative phosphorylation sites targeted by cdk5 and CamKII. Phosphopeptide database (see Table S1) was interrogated using the GKAP sequence; the resulting GKAP phosphoepitopes were annotated using two kinase-target prediction software (Scansite and Phosida). (TIF) [file pone.0023097.s004.tif]

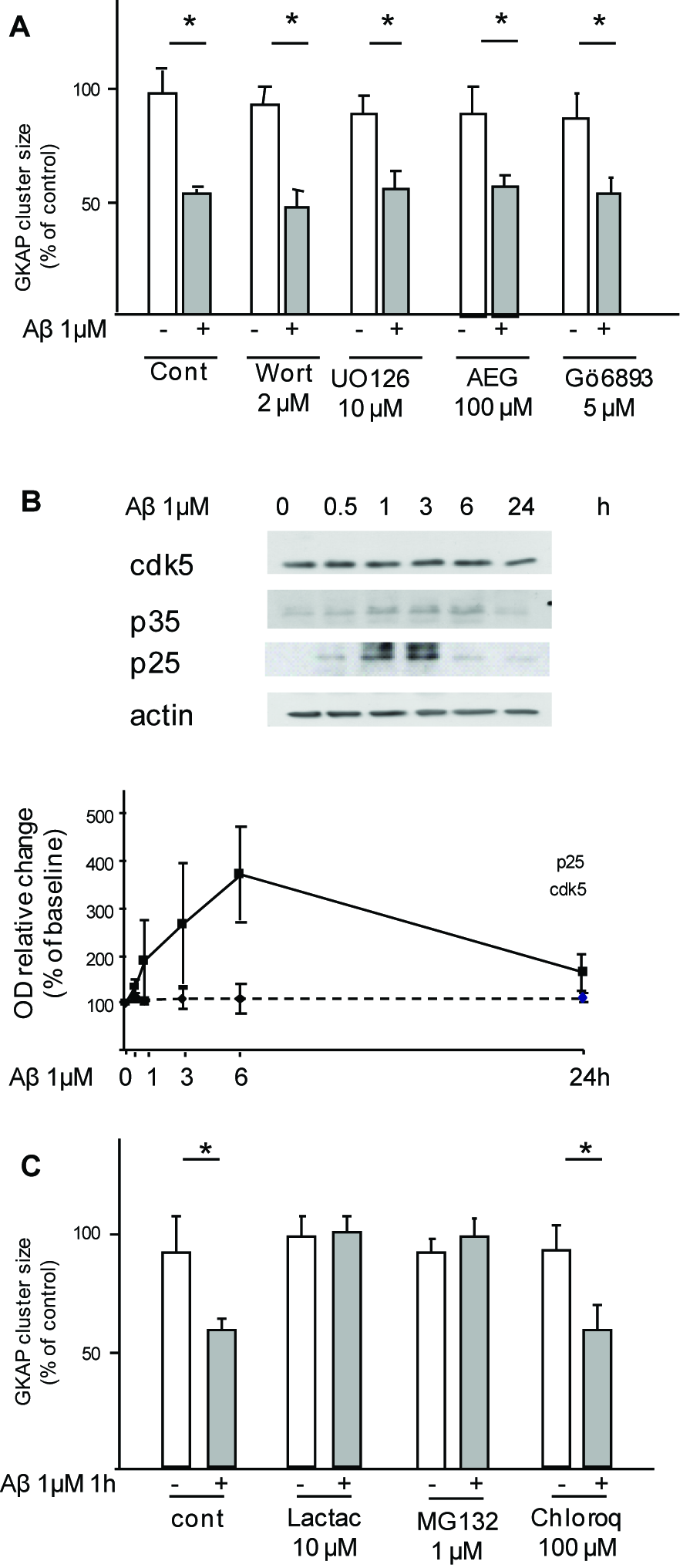

Supplement: Figure S5 — Aβ-induced GKAP degradation does not require PI-3K, ERK, Jnk and PKC activity, but depends on proteasome activity. (A) Rat frontal neurons were pretreated with PI-3K inhibitor Wortmannin (2 µM), ERK inhibitor UO126 (10 µM), Jnk inhibitor (AEG 100 µM) or PKC inhibitor Gö6893 (5 µM) before being exposed to Aβ (1 µM, 1 h). All inhibitors were ineffective (51.5±8.1 Wort+Aβ vs Wort alone, p<0.05; 59.6±9.2 UO+Aβ vs UO alone, p<0.05; 61.7±7.2 AEG+Aβ vs AEG alone, p<0.05; 67.3±5.5 Gö+Aβ vs Gö alone, p<0.05). (B) Rat frontal cortical neurons were treated with Aβ (1 µM) for 0.5, 1, 3, 6 or 24 h. The levels of cdk5 and of the cdk5 activator p35 and the truncated p25 were assessed in whole-cell lysate. Cdk5 levels were not significantly affected by Aβ (111.3±11.4%, 122.2±33.0%, 110.4±23.1%, 109.2±32.3%, 109.4±9.4% at 0.5, 1, 3, 6, and 24 h timepoints, respectively; p>0.05), whereas p35 levels were significantly increased, peaking at 3 h (135.2±16.0%, 216.4±18.9%, 247.8±49%, 176.3±7.2%, 0.86±0.42% at 0.5, 1, 3, 6, and 24 h timepoints) (C) Aβ-induced GKAP degradation requires proteasomal activity. Rat prefrontal neurons were pretreated with the proteasome inhibitors MG132 (1 µM) or lactacysthine (10 µM) or the lysosome pathway blocker chloroquine (100 µM) before being exposed to Aβ (1 µM, 1 h). Aβ effect on GKAP cluster size was completely prevented by MG132 and lactacysthine treatment (107.5±8.3% MG+Aβ vs MG alone, p>0.05; 109.3±7.4%; lacta+Ab vs lacta alone, p>0.05) whereas chloroquine was ineffective (65.4±11.1%, chloroquine+Aβ vs Aβ alone, p<0.05). (TIF) [file pone.0023097.s005.tif]
