# Supplementary material for: CDK5 Is Essential for Soluble Amyloid β-Induced Degradation of GKAP and Remodeling of the Synaptic Actin Cytoskeleton
Source: PLoS One. 2011 Jul 29;6(7):e23097. doi: 10.1371/journal.pone.0023097 (PMC3146526; doi:10.1371/journal.pone.0023097)
Supplement: Table S1 — In silico analysis identifies kinase candidates for the regulation of GKAP. We interrogated a database containing proteomic data regarding post-translational modifications of synaptic proteins (available at http://www.phosphosite.org/) using the reference sequence of SAPAP1 as query, 38 phospho-peptides were retrieved. The phosphopeptides were annotated using Scansite and Phosida phosphorylation site prediction algorithm. Scansite assigned 16 out of 38 phospho-sites to specific kinases; Phosida identified 28 out of 38 potential kinase-phosphosite relationships. Kinases potentially able to phosphorylate GKAP included cdk5, CaMKII, GSK3, PKA, PKC, Akt, and CK. Seven sites were independently confirmed by the two algorithms as potential substrates of cdk5 (according to SAPAP1 reference sequence S134, S403, S437), CaMKII (S666), PKA (S381). For 5 peptides, no annotation was provided by any software. (DOCX) [file pone.0023097.s006.docx]

Table 1

In silico annotation of GKAP phosphorylated sites.

| Phosphorylated aminoacid (position in GKAP sequence/SAPAP1 sequence) | Phosphopeptide sequence | Phosida annotation | Scansite annotation |
| --- | --- | --- | --- |
| S26/352 | CRRMRSGSYIKANGD | CAMKII/AKT | PKC/PKA |
| S36/362 | KAMGDEDSGDSDTSP | ? | CK2 |
| S39/365 | GDEDSGDSDTSPKPS | CK1 | CK1 |
| S55/381 | KVAARRESYLKATQP | PKA/CAMKII | PKA |
| T60/386 | RESYLKATQPSLTEL | NEK6 | ? |
| S63/389 | YLKATQPSLTELTTL |  |  |
| T65/391 | KATQPSLTETTLLKI | ? | ? |
| S77/403 | LKISNEHSPKLQIRS | CDK5 | CDK5 |
| S86/412 | KLQIRSHSYLRAVSE | CAMKII | ? |
| S92/418 | HSYLRAVSEVSINRS | CAMKII | ? |
| S95/421 | LRAVSEVSINRSLDS | CK1/GSK3 | ? |
| S100/425 | SEVSINRSLDSLDPA | CK1 | ? |
| S103/428 | SINRSLDSLDPAGLL | CK1 | ? |
| T110/436 | LDPAGLLTSPKFRSR | ? | ? |
| S111/437 | DPAGLLTSPKFRSRN | CDK5 | CDK5 |
| S120/446 | KFRSRNESYMRAMST | CK1/CAMKII | PKC |
| S123/499 | CFRMRSHSYVRAIEK | CAMKII/AKT | AKT/PKA |
| S183/509 | RAIEKGCSQDDECVS | PKD | ? |
| S190/516 | SQDDECVSLRSSSPP | GSK-3 | ? |
| T199/525 | RSSSPPRTTTTVRTI | ? | ? |
| T200/526 | SSSPPRTTTTVRTIQ | PKA | ? |
| T210/536 | VRTIQSSTGVIKLSS | ? | ? |
| T226/552 | VEVSSCITTYKKTPP | CK1 | ? |
| T227/553 | EVSSCITTYKKTPPP | ? | PKC |
| S279/605 | SQSGLSNSTESLDSM | NEK6 | ? |
| T280/606 | QSGLSNSTESLDSMK | CK1 | ? |
| S282/608 | GLSNSTESLDSMKAL | CK1 | ? |
| S285/611 | NSTESLDSMKALTAA | CK1 | ? |
| S340/666 | FKKNRCLSIGIQVDD | CAMKII | CAMKII |
| S364/691 | KTSSKFQSVGVQVEE | CK1 | ? |
| S621/947 | APLIRERSLESSQRQ | CAMKII/GSK3 | PKC/PKA |
| S647/973 | AASVRQNSATESAES | CAMKII/GSK3 | PKC |
| S649/975 | SVRQNSATESAESIE | ? | ? |
